# Comparative Mitogenomic Analysis of Damsel Bugs Representing Three Tribes in the Family Nabidae (Insecta: Hemiptera)

**DOI:** 10.1371/journal.pone.0045925

**Published:** 2012-09-28

**Authors:** Hu Li, Haiyu Liu, Fan Song, Aimin Shi, Xuguo Zhou, Wanzhi Cai

**Affiliations:** 1 Department of Entomology, China Agricultural University, Beijing, China; 2 Department of Entomology, University of Kentucky, Lexington, Kentucky, United States of America; BiK-F Biodiversity and Climate Research Center, Germany

## Abstract

**Background:**

Nabidae, a family of predatory heteropterans, includes two subfamilies and five tribes. We previously reported the complete mitogenome of *Alloeorhynchus bakeri*, a representative of the tribe Prostemmatini in the subfamily Prostemmatinae. To gain a better understanding of architecture and evolution of mitogenome in Nabidae, mitogenomes of five species representing two tribes (Gorpini and Nabini) in the subfamily Nabinae were sequenced, and a comparative mitogenomic analysis of three nabid tribes in two subfamilies was carried out.

**Methodology/Principal Findings:**

Nabid mitogenomes share a similar nucleotide composition and base bias, except for the control region, where differences are observed at the subfamily level. In addition, the pattern of codon usage is influenced by the GC content and consistent with the standard invertebrate mitochondrial genetic code and the preference for A+T-rich codons. The comparison among orthologous protein-coding genes shows that different genes have been subject to different rates of molecular evolution correlated with the GC content. The stems and anticodon loops of tRNAs are extremely conserved, and the nucleotide substitutions are largely restricted to TψC and DHU loops and extra arms, with insertion-deletion polymorphisms. Comparative analysis shows similar rates of substitution between the two rRNAs. Long non-coding regions are observed in most Gorpini and Nabini mtDNAs in-between *trnI*-*trnQ* and/or *trnS2*-*nad1*. The lone exception, *Nabis apicalis*, however, has lost three tRNAs. Overall, phylogenetic analysis using mitogenomic data is consistent with phylogenies constructed mainly form morphological traits.

**Conclusions/Significance:**

This comparative mitogenomic analysis sheds light on the architecture and evolution of mitogenomes in the family Nabidae. Nucleotide diversity and mitogenomic traits are phylogenetically informative at subfamily level. Furthermore, inclusion of a broader range of samples representing various taxonomic levels is critical for the understanding of mitogenomic evolution in damsel bugs.

## Introduction

Insect mitochondrial genome (mitogenome) typically consists a single circular molecule that is 14–20 kb long and usually contain 13 protein-coding genes (PCGs), 22 transfer RNAs (tRNAs), two ribosomal RNAs (rRNAs) (the large and small ribosomal subunits), and one or more non-coding (NC) regions (also referred to as the control region, CR) with essential regulatory elements for transcription and replication [1], [2]. Notable exceptions are represented by the fragmented mtDNA of some sucking lice and booklice [3]–[5]. Several advantages including simple genomic organization, high rates of evolution, and (almost) unambiguous orthology have made the mitogenomes good models for molecular evolution and abundant molecular markers for phylogeographic and phylogenetic studies at various taxonomic levels [6]–[10]. Complete mitogenomes are not only more informative than individual genes, but also provide a suite of genome level characters, such as the relative position of different genes, RNA secondary structures and models of control of replication and transcription. Mitogenomic sequencing and subsequent analysis of the 13 PCGs have increased dramatically in the past decade and the utility of mtDNAs for phylogenetic inference at various taxonomic levels has therefore been aggressively exploited [8].

Nabidae, also called damsel bugs, is a relatively small family of Heteroptera with approximately 20 genera and 500 species [11] and comprise of two subfamilies Prostemmatinae and Nabinae, and five tribes [12]. Most members of Prostemmatinae appear more stout-bodied and occasionally posses distinctive red and black color patterns. They are ground-living with enlarged and strong forelegs and appear to prey exclusively on other insects. In contrast, the Nabinae are elongate and of drab coloration, live on plant, and prey on small arthropods with simple forelegs [12], [13]. The distinctive subfamily level differences make the damsel bugs an ideal group to study the evolution of mitogenomes.

The complete mitogenome of *Alloeorhynchus bakeri*, a representative of the tribe Prostemmatini in the subfamily Prostemmatinae has been reported previously [14]. In this study, three complete and two nearly complete mitogenomes from two tribes, Gorpini and Nabini, in the subfamily Nabinae were sequenced. Overall, six nabid mitogenomes representing two subfamilies, three tribes, and four genera were used in the comparative analysis to: 1) assess evolutionary traits of mitogenomes from three tribes, and 2) explore the phylogenetic utility and limits of mitogenomic data at lower taxonomic levels, specifically subfamily and generic levels.

## Results and Discussion

### Genome Organization

In this study, four complete and two nearly complete mitogenomes of damsel bugs representing three tribes and two subfamilies were compared. Four complete mitogenomes were from *Alloeorhynchus bakeri* Harris [14], *Nabis apicalis* Matsumura, *Gorpis annulatus* Paiva, and *Gorpis humeralis* (Distant), and two nearly complete mitogenomes were from *Himacerus apterus* (Fabricius) and *Himacerus nodipes* (Hsiao). Regions that we failed to sequence were located in or around gene *nad2* and CR. Several factors including notable base bias, highly repeated regions, and stable secondary structures could result in the unsuccessful sequencing of the CR.

All six mitogenomes shared overlapping genes, typically 10 to 13 gene overlaps; the highest number (17) of gene overlaps was found in *A. bakeri* and the fewest number (10) was found in *G. annulatus*. The amount of overlap between genes was typically between 1 and 14 bp. Two pairs of overlapping genes were common to all six mitogenomes: *atp8*-*atp6* and *nad4*-*nad4L*, and shared nearly the same 7 bp sequence (ATGATAA), with the exception of *A. bakeri* (ATGATAG overlap between *nad4* and *nad4L*). This is a common feature in heteropteran mtDNAs, as well as in other arthropods [15].

**Figure 1:**
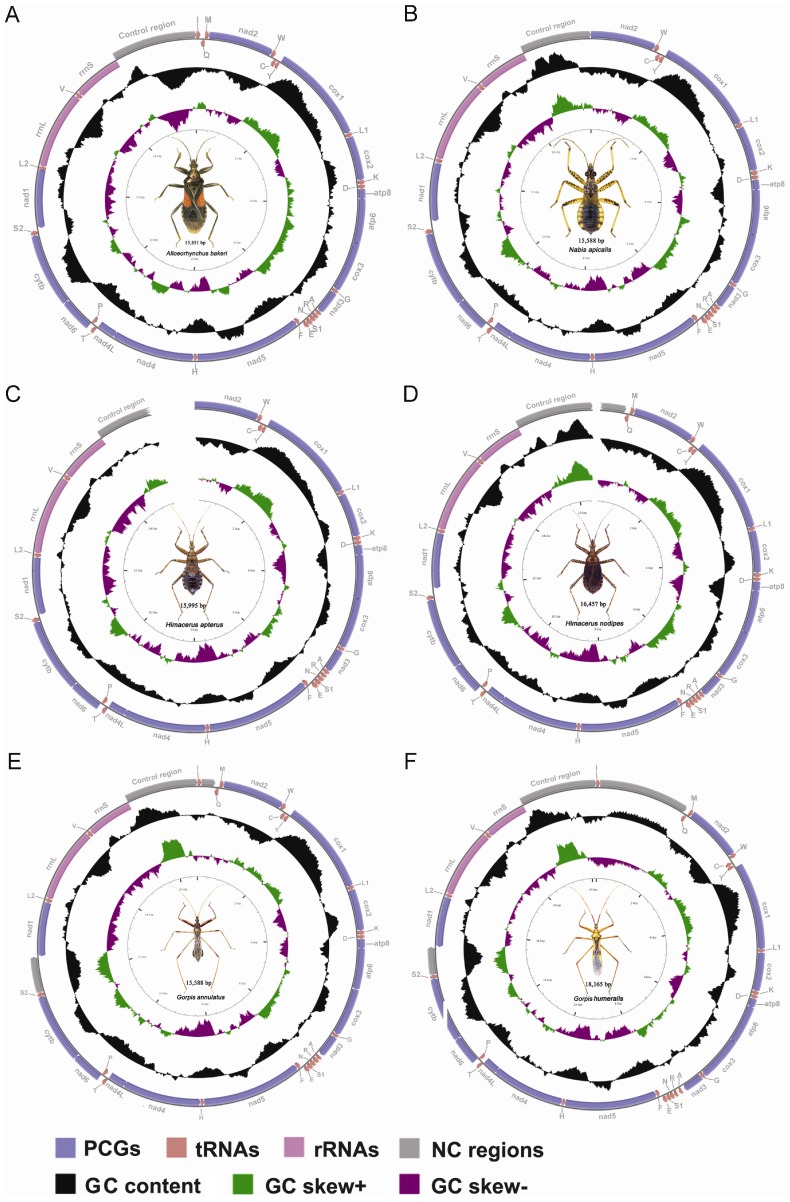
Mitochondrial maps of six damsel bugs. Direction of gene transcription is indicated by arrows. PCGs are shown as blue arrows, rRNAs as purple arrows, tRNAs as red arrows and large NC regions (>100 bp) as cyan rectangles. tRNAs are labeled according to single-letter IUPAC-IUB abbreviations (L1: UUR; L2:CUN; S1:AGN; S2:UCN). The GC content is plotted using a black sliding window, as the deviation from the average GC content of the entire sequence. GC-skew is plotted as the deviation from the average GC-skew of the entire sequence. Ticks in the inner cycle indicate the sequence length.

Consistent with other published heteropteran mitogenomes, the six nabid mitogenomes contained all 13 PCGs and both rRNAs in the same order and direction of the hypothesized ancestral arthropod mitogenome [16] (Figure 1). Most complete mitogenomes typically had 22 tRNAs, however, only 19 tRNAs were detected in *N. apicalis*, with loss of the gene cluster *trnI-trnQ-trnM*. All detected tRNAs shared identical architectures at the tRNA loci, similar to most heteropterans.

Most PCGs started with ATN start codons and stopped with TAA/TAG termination codons or truncated termination codons TA or T which were presumed to be completed via post-transcriptional polyadenylation [17]. Gene *cox1* and *nad1* has been found to employ TTG or GTG as a start codon in some species of Hemiptera, Hymenoptera, Coleoptera and Lepidoptera, thus minimizing intergenic spacing and avoiding overlaps with adjacent genes [18]–[20]. We discovered TTG start codons in *cox1* gene for most sequenced damsel bugs and GTG start codon in *nad1* gene for *G. humeralis* (Table S1).

### Nucleotide Composition and Codon Usage

All analyzed nabid mitogenomes were consistently AT biased, with values from 72.6% to 76.9% and, on average, displayed positive AT- and negative GC-skews of the coding strand (Figure S1A). The J-strand of PCGs and rRNAs had a negative AT-skew in all species, that of tRNAs had a positive AT-skew (Figure S1B). The tRNAs and rRNAs had a positive GC-skew, and the 3rd codon positions had a negative GC-skew (Figure S1C). The G content was almost equal to C content in PCGs in all species. All sequences showed strong consistent base bias, except the CR showed a large variation in AT- and GC-skews.

The pattern of codon usage in all analyzed damsel bug mitogenomes was consistent with the standard invertebrate mitochondrial genetic code and the preference for A+T-rich codons. They shared the similar codon usage bias an effective number of codons (ENC) [21] equivalent to 38.45±3.58 and codon bias index (CBI) [22] equivalent to 0.65±0.07. Synonymous codons ending with A or T were clearly preferred (81.5 to 89.8% for individual species; 86.2% on average), and the most frequently used codons were AUA, UUA, AUU, UUU, UAU and AAU (Figure S2). Some G+C-rich codons were not used in some species, such as CGC in *N. apicalis*, UCG and GCG in *H. apterus*, and CUC, GCG and GGC in *H. nodipes* (Table S2).

**Figure 2:**
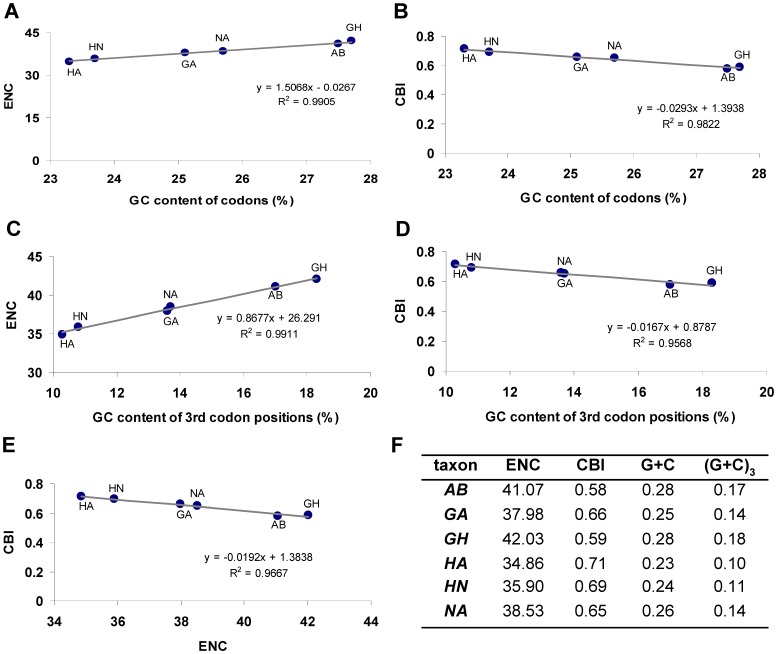
Evaluation of codon bias across six nabid mtDNAs. ENC, effective number of codons (out of a maximum of 61) [21]; CBI, codon bias index [22]; G+C, GC content of codons; (G+C)_3_, GC content of 3rd codon positions.

The correlations among CBI, ENC, GC content of all codons, and GC content of the 3rd codon positions in all sequenced damsel bug mitogenomes were analyzed (Figure 2). A positive correlation was observed between ENC and GC content of all codons (R^2^ = 0.99) (Figure 2A) and the 3rd codon positions (R^2^ = 0.99) (Figure 2C). Furthermore, a negative correlation was observed between CBI and GC content of all codons (R^2^ = 0.96) (Figure 2B), GC content of the 3rd codon positions (R^2^ = 0.96) (Figure 2D) and ENC (R^2^ = 0.97) (Figure 2E). These results are consistent with prevailing neutral mutational theories positing that genomic GC content is the most significant factor in determining codon bias among organisms [23]–[25].

### Evolutionary Rate

In view of the evolutionary forces acting on the mitochondrial PCGs of three nabid tribes, the average rate of non-synonymous substitutions (Ka), the average rate of synonymous substitutions (Ks), and the average ratio of Ka/Ks were calculated for each PCG, respectively [26]. The observed average Ka/Ks ratios were consistently lower than one, increasing from 0.07 for *cox1* to 0.93 for *atp8* (Table S3). The uniformly low values of Ka/Ks ratios for *cox1–3* and *cytb* (Ka/Ks <1) indicate strong evolution constraints in cytochrome *c* oxidase [27], [28] and also suggest a strong purifying selection in the six species of nabids. The variation of GC content probably caused the different evolutionary patterns among genes, and a negative correlation was observed between the Ka/Ks and the GC content of mitochondrial PCGs (R^2^ = 0.85). This result is highly consistent with previous findings in true bugs [14], [29].

**Figure 3:**
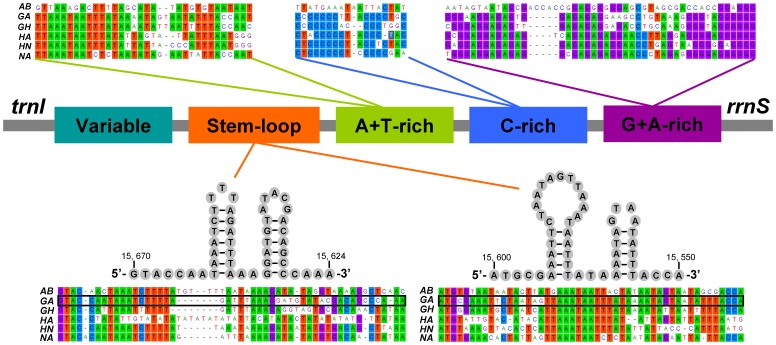
Organization of the control region in six nabid mtDNAs. The colorized panes indicate the structural elements in the CR. The alignment of the primary sequences and the predicated secondary stem-loop structures are shown, respectively, above and below.

### Transfer RNAs

The tRNAs closest to the CR (ie., *trnI*, *trnQ* and *trnM*), which is one of the starting points for mtDNA replication, were not detected in the complete mitogenome of *N. apicalis* (Figure 1). Among tRNAs, only *trnS1* did not exhibit the typical clover leaf secondary structure, due to the loss of the DHU stem (Figure S3). In general, the loss of the DHU stem in *trnS1* was a common feature in metazoan mtDNAs [30], and is almost always presents in Heteroptera [20], [29].

The *trnA*, *trnR*, *trnN*, *trnQ*, *trnK*, *trnS1*, *trnS2*, and *trnV* showed more mismatches in their stems (Figure S3). Mismatches were located mostly in the acceptor and anticodon stems with a single exception represented by *trnV*, in which the mismatch was on the TψC stem. Mismatches observed in tRNAs are corrected through the RNA-editing mechanisms that are well known for arthropod mtDNA [31]–[33].

In most nabid tRNAs, the stems and anticodon loops were extremely conserved, and the nucleotide substitutions were largely restricted to TψC and DHU loops and extra arms, with obvious insertion-deletion polymorphisms (Figures S4, S5). The presence of indels polymorphisms at different taxonomic levels underscores the potential phylogenetic value of tRNA sequences, especially when secondary structures are taken into account [30], [34], [35].

### Ribosomal RNAs

Genes for the small and large subunit ribosomal RNAs (*rrnS* and *rrnL*) were adjacent on the same strand, interleaved by a single *trnV*, in the analyzed damsel bug mitogenomes. Both rRNAs of *N. apicalis* were the largest (1, 289 bp in *rrnL* and 820 bp in *rrnS*), while those in *A. bakeri* were the smallest (1, 252 bp in *rrnL* and 790 bp in *rrnS*) (Table S1). The size differences in both ribosomal subunits were not distinct among different species, especially in the subfamily Nabinae (1, 283±6 bp in *rrnL* and 812±6 bp in *rrnS*).

With the addition of five newly sequenced damsel bugs, the mitochondrial rRNA secondary structures were compared in three tribes. All of rRNA genes conformed to the secondary structure models proposed for *A. bakeri* [14] and other insects [36]–[39]. The overall structure of *rrnL*s, including five domains and 44 helices, were largely consistent among the analyzed damsel bugs. The multiple alignment of *rrnL*s extended over 1, 345 positions and contained 964 conserved (71.7%), 85 indels (6.3%) and 296 nucleotide substitutions (22.0%). Nucleotide variability was unevenly distributed among domains and helices, mainly in domains I and II.

Similarly, the secondary structures of *rrnS* were also largely consistent, including three domains and 27 helices. The multiple alignments spanned 849 positions and contained 608 conserved (71.6%), 59 indels (6.9%) and 182 nucleotide substitutions (21.4%). Nucleotide variability was unevenly distributed among domains and helices, mainly in domain I and the large loop between helices H577 and H673.

Comparative studies showed similar rates of substitution between two rRNAs (Figure S6). Several helices (H235, H589, H837, and H2077 in *rrnL,* and H39, H47, H367, H1241, and H1303 in *rrnS*, respectively) with high variability at the primary sequence level, showed conserved secondary structures. The phylogenetic value of structural characteristics of these helices is intriguing, suggesting the necessity of sampling at different taxonomic levels to ensure the validity of phylogenetic relationships inferred by the mitogenomic data.

### Non-coding Regions

The six mitogenomes of Nabidae displayed moderate size variation, most of which could be attributed to the variety of NC regions usually caused by the presence of repetitive elements. The NC regions of six nabid mitogenomes were summarized in Table S4; the size varied from 1, 198 bp (*N. apicalis*, the shortest) to 3, 567 bp (*G. humeralis*, the largest). The proportion of NC regions appeared high in the mtDNAs of *N. apicalis* and *G. humeralis*, varying from 7.7 to 19.6%. Three distinct large NC regions were identified in the following gene pairs: *trnI*-*trnQ*, *trnS2*-*nad1*, and *rrnS-trnI*.

The NC region located between *trnI* and *trnQ*, appeared to be common in the *Gorpis* and *Himacerus* mitogenomes, varying in length from 221 to 1,539 bp. Tandem repeats were detected in this region of *G. humeralis* (1, 442 bp and six copies) and *H. nodipes* (540 bp and four copies) (Table S5). This region was also present in other heteropteran mtDNAs even though the nucleotide sequence could be divergent among species [29], [40]. Several arrangements at this genomic location are worth noting: (1) the gene cluster *trnI*- *trnQ* -*trnM* was missing in the mtDNA of *N. apicalis*; (2) *trnI* and *trnQ* were reversed in the flat bug *Neuroctenus parus* [29]; and (3) the gene order *trnM*-*trnI*-*trnQ* and the NC regions between *trnQ* and *nad2* were present in almost all sequenced lepidopteran mtDNAs [37], [41]–[44]. Further investigation with a broader taxon sampling within Heteroptera is necessary to assess if this genomic location is related to the replication of mtDNA.

The NC region inserted between *trnS2* and *nad1*, was present in all sequenced nabid mtDNAs, varying in length from 15 to 584 bp. Tandem repeats were detected in this location of *G. annulatus* (582 bp, 4 copies) and *G. humeralis* (467 bp, 3 copies) (Table S5). This intergenic spacer is also present in all partial or fully sequenced neuropterid species and other insect mtDNAs [30], and contains the binding site for the DmTFF bidirectional transcription termination factor [45].

The NC region located between *rrnS* and *trnI*, coincided with the A+T-rich region, also called the CR, including the origin of replication and promoters for transcription initiation [2], [46]–[48]. The CRs of three nabid tribes were longer than 1 kb, with high rates of nucleotide substitution and indels, and a variable number of tandem repeats.

Comparison of the CR sequences of six nabid mtDNAs revealed few relevant short blocks. The pattern of sequence conservation is in agreement with previous reports, indicating that CRs of three nabid tribes (especially the five species in the subfamily Nabinae) belong to the group 2 insect-control region [46], with the short conserved blocks, as well as the conserved secondary structures.

The following structural elements have been identified from comparative analysis of the CR sequences of six nabid species belonging to two subfamilies (Figure 3).

1. A G+A-rich sequence block was conserved at the 3′-end of the CR (near to *rrnS*). The overall low similarity of the primary nucleotide sequences between Nabinae and Prostemmatinae suggests that they are taxon-specific.
2. Upstream of the conserved G+A-rich sequence block was a conserved C-rich sequence block in Gorpini and Nabini, but not presented in Prostemmatini.
3. An A+T-rich sequence block was identified upstream of C-rich sequence block.
4. The stem-loop structures were conserved in three tribes, and are potentially associated with the second strand replication origin [47]–[51].
5. One variable domain concluded the rest of the region and was highly variable in both nucleotide sequence and length. Tandem repetition was observed in this region.

Comparative analysis of the structural organization of CRs among three nabid tribes revealed some highly conserved sequences, but also revealed distinct differences between the two phylogenetically-distant damsel bug subfamilies. The utility of the CR as a phylogenetic marker should be most effective at lower taxonomic ranks (subfamily level and below).

### Molecular Phylogeny

**Figure 4:**
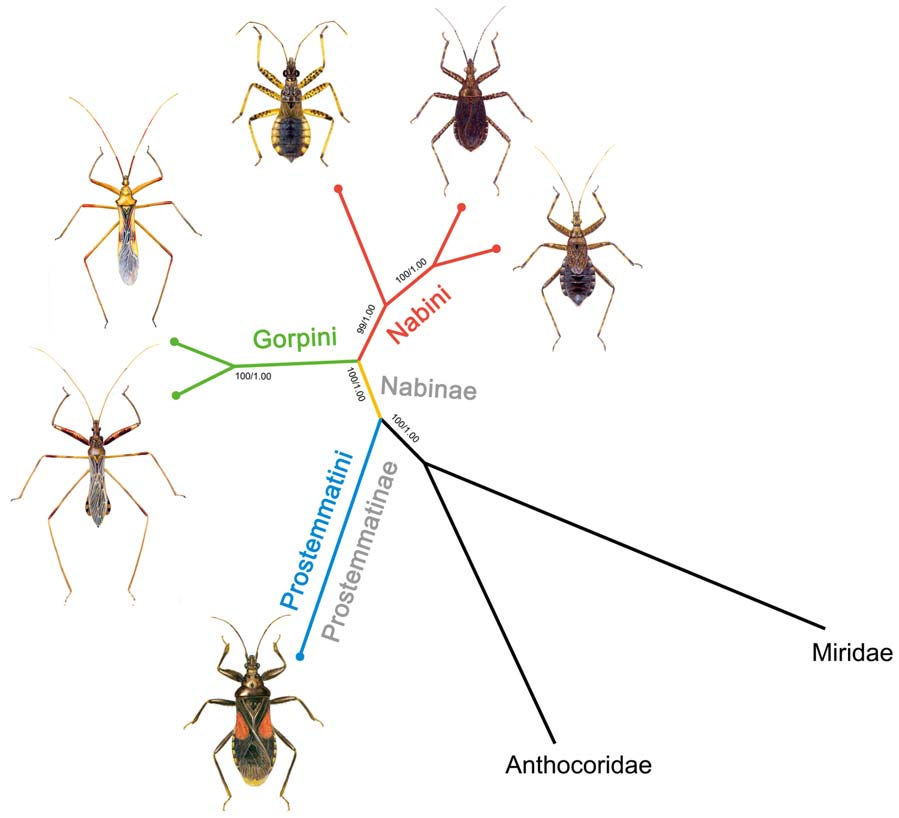
Molecular phylogenetic tree of six damsel bugs. Phylogenetic analysis was based on all mitochondrial genes (excluding *trnI*, *trnQ* and *trnM*). The tree was rooted with two outgroup taxa (*O. niger* and *L. lineolaris*). Numbers close to the branching points are percentages of ML bootstrap support values (left) and Bayesian posterior probabilities (right).

Phylogenetic analysis was performed on a concatenated nucleotide dataset of all PCGs, rRNAs and 19 tRNAs (excluding *trnI*, *trnQ* and *trnM*). The trees from the ML and Baysian analysis shared identical topologies and high node support values (Figure 4). The results indicated the sister-group relationships between *A. bakeri* (subfamily Prostemmatinae) and the other five species (subfamily Nabinae). Within the subfamily Nabinae the tribes Gorpini and Nabini were seen as sister groups. Within the tribe Nabini, the genus *Nabis* formed a sister group with the remaining *Himacerus* species.

**Table 1:** List of the species included in the present study.

Classification	Species	Accession No.	Reference
Anthocoridae	*Orius niger*	NC_012429	[40]
Miridae	*Lygus lineolaris*	EU401991*	Direct submission
Nabidae			
Prostemminae: Prostemmatini	*Alloeorhynchus bakeri*	HM235722	[14]
Nabinae: Gorpini	*Gorpis annulatus*	JF907591	Present study
Nabinae: Gorpini	*Gorpis humeralis*	JF927830	Present study
Nabinae: Nabini	*Himacerus apterus*	JF927831*	Present study
Nabinae: Nabini	*Himacerus nodipes*	JF927832*	Present study
Nabinae: Nabini	*Nabis apicalis*	JF907590	Present study

“*”nearly complete mitogenomes. All accession numbers were retrieved from GenBank.

The relationship of three tribes from two subfamilies of Nabidae was well represented by the mitogenome data and was consistent with current phylogeny of the family Nabidae constructed mainly from morphological traits. Nabidae included two subfamilies Prostemmatinae and Nabinae, and five tribes [13]. Prostemmatinae are ground living, more stout-bodied and bright color (red and black), and display the ancestral type of karyotype (high chromosome number) [52]. This subfamily includes two tribes, Phorticini and Prostemmatini, and is considered the basal lineage in Nabidae based on their morphological and ecological traits (Figure S7). Our phylogenetic analysis based on the mitogenome sequence validates the basal position of Prostemmatini among three tribes. In addition, comparative analysis of mtDNAs also provided a genome level perspective that the architectures of Gorpini and Nabini mtDNAs were more closely related and similar, in comparison to the Prostemmatini. These similarities include having an apparently large NC region at *trnI*-*trnQ* and/or *trnS2*-*nad,* and three conserved sequence blocks (A+T-rich, C-rich and G+C-rich block) in CR (Figure S7).

Overall, nucleotide diversity and genomic traits make the mitogenomes a promising marker for resolving phylogenetic relationships at various taxonomic levels. In this comparative mitogenomic analysis, our understanding of the phylogeny of Nabidae is improved by the inclusion of representatives from three tribes and the two subfamilies.

### Summary

Sequenced mitogenomes of three nabid tribes shared strong consistent nucleotide composition and base bias, except the CR showed large variation in AT- and GC-skews. The pattern of codon usage was influenced by the genomic GC content and consistent in three tribes with the standard invertebrate mitochondrial genetic code and the preference for A+T-rich codons. The comparison among orthologous PCGs showed that different genes have been subject to different rates of molecular evolution that are correlated with the GC content.

In most nabid tRNAs, stems and anticodon loops were extremely conserved, and nucleotide substitutions were largely restricted to TψC and DHU loops and extra arms, with apparent insertion-deletion polymorphisms. Comparative studies showed similar rates of substitution between the two rRNAs and some helices with high variability at the primary sequences shared good structural covariation. Architecture of six nabid mitogenomes exhibited substantial differences between the two subfamilies. Large NC regions at *trnI*-*trnQ* and/or *trnS2*-*nad1* were present in all known nabid mtDNAs except *N. apicalis* in which *trnI*, *trnQ*, and *trnM* were missing. Comparative analysis of CRs revealed some highly conserved sequence blocks as well as distinct differences in different tribes and subfamilies. Overall, the phylogenetic analysis using mitogenomes are consistent with the current phylogeny of the family Nabidae derived mainly from morphological traits. Our study has demonstrated that nucleotide diversity and mitogenomic traits have the potential to provide insights of phylogenetic relationships at the subfamily level.

## Materials and Methods

### Ethics Statement

No specific permits were required for the insect collected for this study. The insect specimens were collected from road side vegetation by sweeping. The field collections did not involve endangered or protected species. The species in the family of Nabidae are common insects and are not included in the “List of Protected Animals in China”.

### Samples and DNA Extraction

All species were collected in China between 2007 and 2009 (Table S6). All collections were initially preserved in 95% ethanol in the field, and transferred to −20°C for long-term storage at the China Agricultural University (CAU). For each species, the genomic DNA was extracted from one male adult’s muscle tissues of the thorax using a CTAB-based protocol [53].

### PCR Amplification and Sequencing

The mitogenome was generated by amplification of overlapping PCR fragments (Table S7) using a range of universal insect mitochondrial primers [8]. Species-specific primers were designed based on the sequenced fragments to bridge gaps. PCR and sequencing reactions were conducted following [14], [20], [39].

### Annotation and Bioinformatics Analysis

PCGs and rRNAs were initially identified using BLAST searches in GenBank and subsequently by alignment with genes of other true bugs. tRNAs were identified by tRNAscan-SE Search Server v.1.21 [54]. Some tRNA genes that could not be determined by tRNAscan-SE were determined in the unannotated regions by sequence similarity to tRNAs of other heteropterans. The base composition, codon usage, and nucleotide substitution were analyzed with Mega 5.0 [55]. AT-skew = (A−T)/(A+T) and GC- skew = (G−C)/(G+C) were used to measure the base-compositional difference between genes [56].

The software packages DnaSP 5.0 [57] was used to calculate the number of synonymous substitutions per synonymous site (Ks), the number of nonsynonymous substitutions per nonsynonymous site (Ka), the effective number of codons (ENC) and codon bias index (CBI) for PCGs.

### Construction of Secondary Structures of RNAs and Non-coding Regions

Secondary structures of the small and large subunits of rRNAs were inferred using models predicted for *A. bakeri* [14] and other species of insects [36]–[39]. Stem-loops were named according to the convention of [36], [37]. Regions lacking significant homology and other non-coding regions were folded using Mfold [58].

### Phylogenetic Analysis

Six nabid species with complete or nearly complete mitogenomes were used in the phylogenetic analysis, representing two subfamilies and three tribes (Table 1). Two species, *Orius niger* from Anthocoridae and *Lygus lineolaris* from Miridae, were selected for outgroup comparisons.

The complete sequences of each gene were used for phylogenetic analysis (excluding stop codons of the PCGs, *trnI*, *trnQ* and *trnM*). All PCGs were aligned based on amino acid sequence alignments in MEGA version 5.0 [55]. The rRNAs and tRNAs were aligned with Clustal X version 2.07 [59]. tRNA alignments were corrected according to secondary structure. Alignments of individual genes were then concatenated as a combined matrix. Model selection was based on Modeltest 3.7 [60] for nucleotide sequences. According to the Akaike information criterion, the GTR+I+G model was optimal for analysis with nucleotide alignments. MrBayes Version 3.2.1 [61] and a PHYML online web server [62], [63] were employed to reconstruct the phylogenetic trees. In Bayesian inference, two simultaneous runs of 3,000,000 generations were conducted for the matrix. Each set was sampled every 1000 generations with a burn-in of 25%. Trees inferred prior to stationarity were discarded as burn- in, and the remaining trees were used to construct a 50% majority-rule consensus tree. In ML analysis, the parameters were estimated during analysis and the node support values were assessed by bootstrap resampling (BP) [64] calculated using 100 replicates.

## Supporting Information

Figure S1 **Nucleotide composition of six nabid mitogenomes.** (A) AT content; (B) AT- skew; (C) GC-skew. The values are shown for the J-strand of the whole genome, its concatenated genetic components (PCGs, tRNAs and rRNAs), 3rd codon positions in PCGs, and the control region (CR). Species are abbreviated as following: *AB*, *A. bakeri*; *NA*, *N. apicalis*; *GA*, *G. annulatus*; *GH*, *G. humeralis*; *HA*, *H. apterus*; *HN*, *H. nodipes*.
(TIF)

Figure S2 **Codon distribution in six nabid mtDNAs.** Numbers to the left refer to the total number of codon. Codon families are provided on the x axis. Most frequently used codons are highlighted in red.
(TIF)

Figure S3 **Inferred secondary structure of tRNA families in six nabid mtDNAs.** The nucleotide substitution pattern for each tRNA family was modeled using as reference the structure determined for *G. annulatus.* The identical nucleotides in all six nabid mtDNAs are showed by grey circles. Not conserved nucleotides are highlighted by blue circles. The tRNAs are labeled with the abbreviations of their corresponding amino acids. Inferred Watson-Crick bonds are illustrated by lines, whereas GU bonds are illustrated by dots.
(TIF)

Figure S4 **Alignment of tRNA families (** ***trnA*** **-** ***trnL1*** **) in six nabid mtDNAs.** The loop regions are highlighted by the black pane.
(TIFF)

Figure S5 **Alignment of tRNA families (** ***trnL2*** **-** ***trnV*** **) in six nabid mtDNAs.** The loop regions are highlighted by the black pane.
(TIFF)

Figure S6 **Nucleotide variation in six nabid mitochondrial rRNAs.**
(TIF)

Figure S7 **Mapping various traits onto the phylogenetic tree of six damsel bugs.** Ecological, morphological and mitogenomic traits are mapped onto the phylogenetic tree inferred from the mitogenomic data.
(TIF)

Table S1 **Structural features of six nabid mitogenomes.**
(DOC)

Table S2 **Codon distribution in six nabid mtDNAs.**
(DOC)

Table S3 **Evolutionary rates of six nabid mitochondrial PCGs.**
(DOC)

Table S4 **Statistics on NC sequences in six nabid mitogenomes.**
(DOC)

Table S5 **Occurrence of tandem repetitions in six nabid mtDNA NC regions.**
(DOC)

Table S6 **Information of five sequenced nabid species included in the present study.**
(DOC)

Table S7 **Primer sequences used in this study.**
(DOC)
