# Supplementary figures and images for: Comparative Mitogenomic Analysis of Damsel Bugs Representing Three Tribes in the Family Nabidae (Insecta: Hemiptera)
Source: PLoS One. 2012 Sep 28;7(9):e45925. doi: 10.1371/journal.pone.0045925 (PMC3461043; doi:10.1371/journal.pone.0045925)

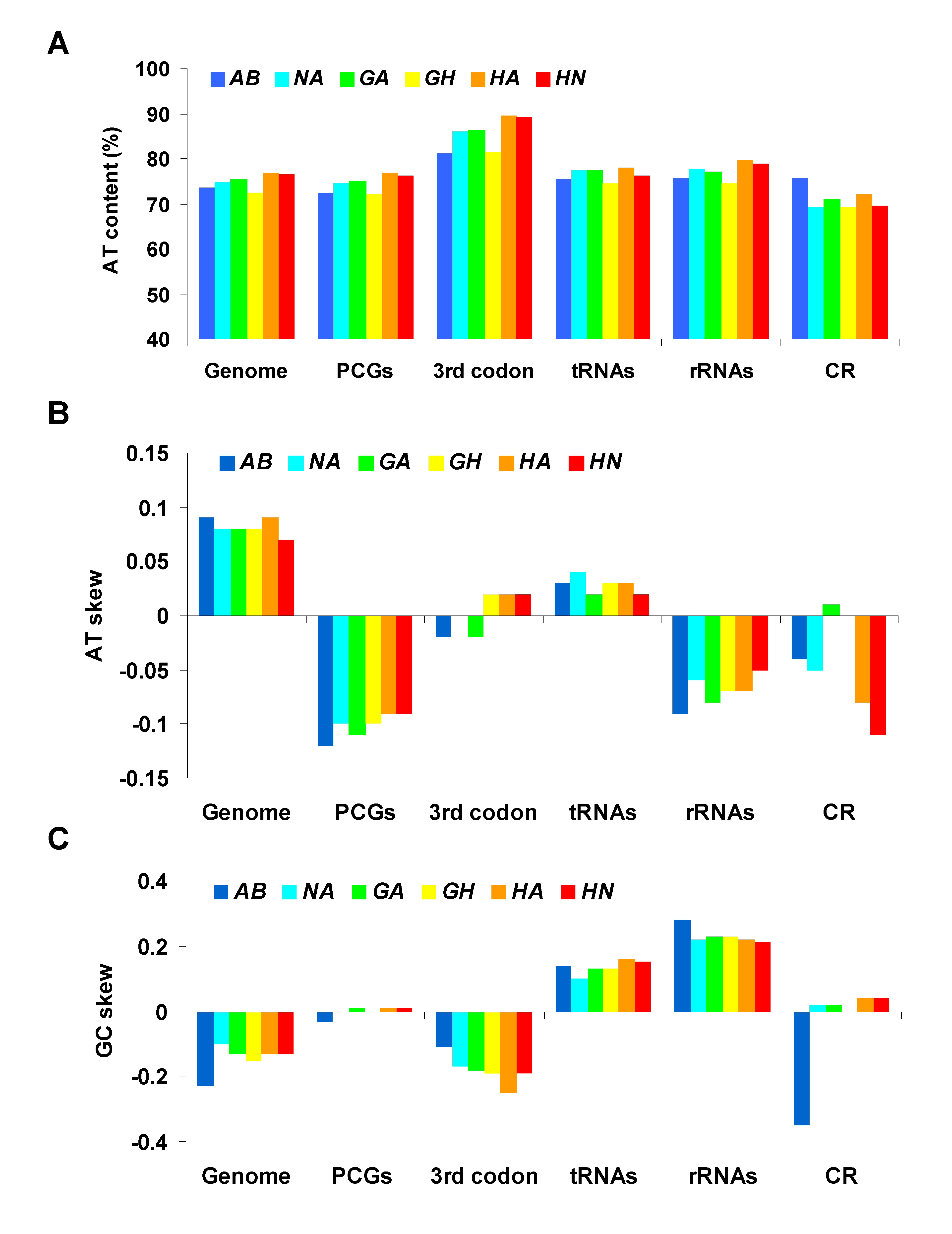

Supplement: Figure S1 — Nucleotide composition of six nabid mitogenomes. (A) AT content; (B) AT- skew; (C) GC-skew. The values are shown for the J-strand of the whole genome, its concatenated genetic components (PCGs, tRNAs and rRNAs), 3rd codon positions in PCGs, and the control region (CR). Species are abbreviated as following: AB, A. bakeri; NA, N. apicalis; GA, G. annulatus; GH, G. humeralis; HA, H. apterus; HN, H. nodipes. (TIF) [file pone.0045925.s001.tif]

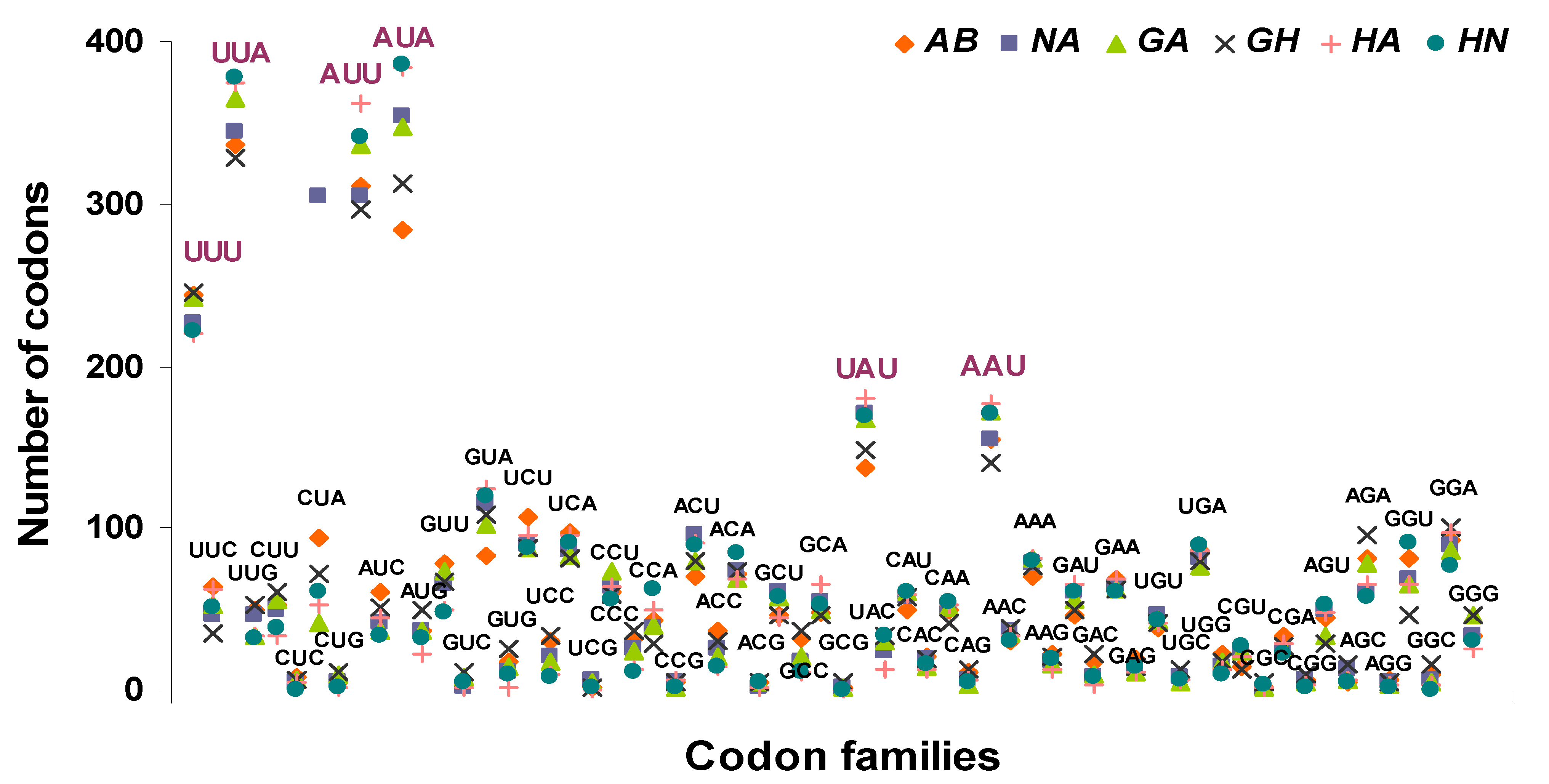

Supplement: Figure S2 — Codon distribution in six nabid mtDNAs. Numbers to the left refer to the total number of codon. Codon families are provided on the x axis. Most frequently used codons are highlighted in red. (TIF) [file pone.0045925.s002.tif]

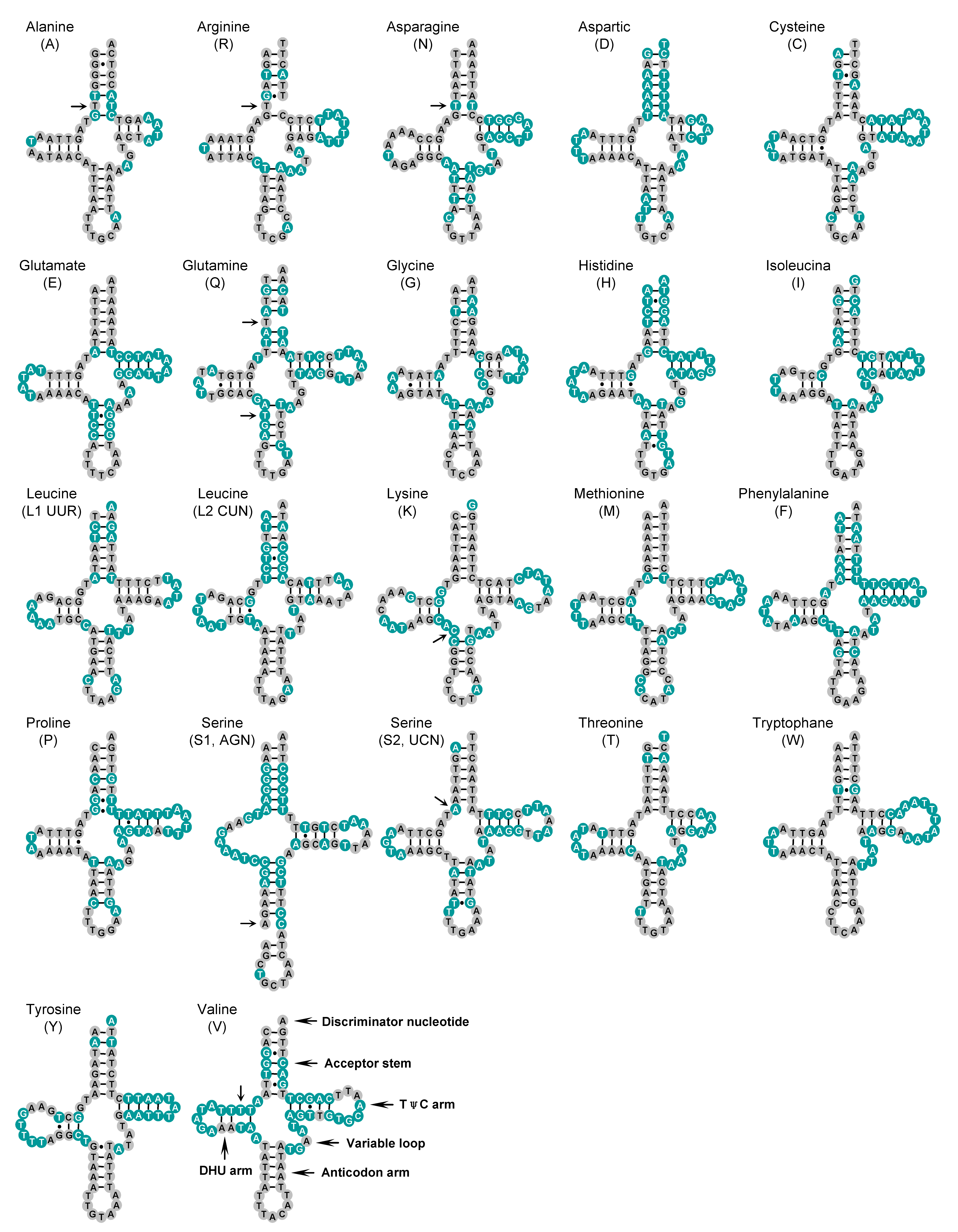

Supplement: Figure S3 — Inferred secondary structure of tRNA families in six nabid mtDNAs. The nucleotide substitution pattern for each tRNA family was modeled using as reference the structure determined for G. annulatus. The identical nucleotides in all six nabid mtDNAs are showed by grey circles. Not conserved nucleotides are highlighted by blue circles. The tRNAs are labeled with the abbreviations of their corresponding amino acids. Inferred Watson-Crick bonds are illustrated by lines, whereas GU bonds are illustrated by dots. (TIF) [file pone.0045925.s003.tif]

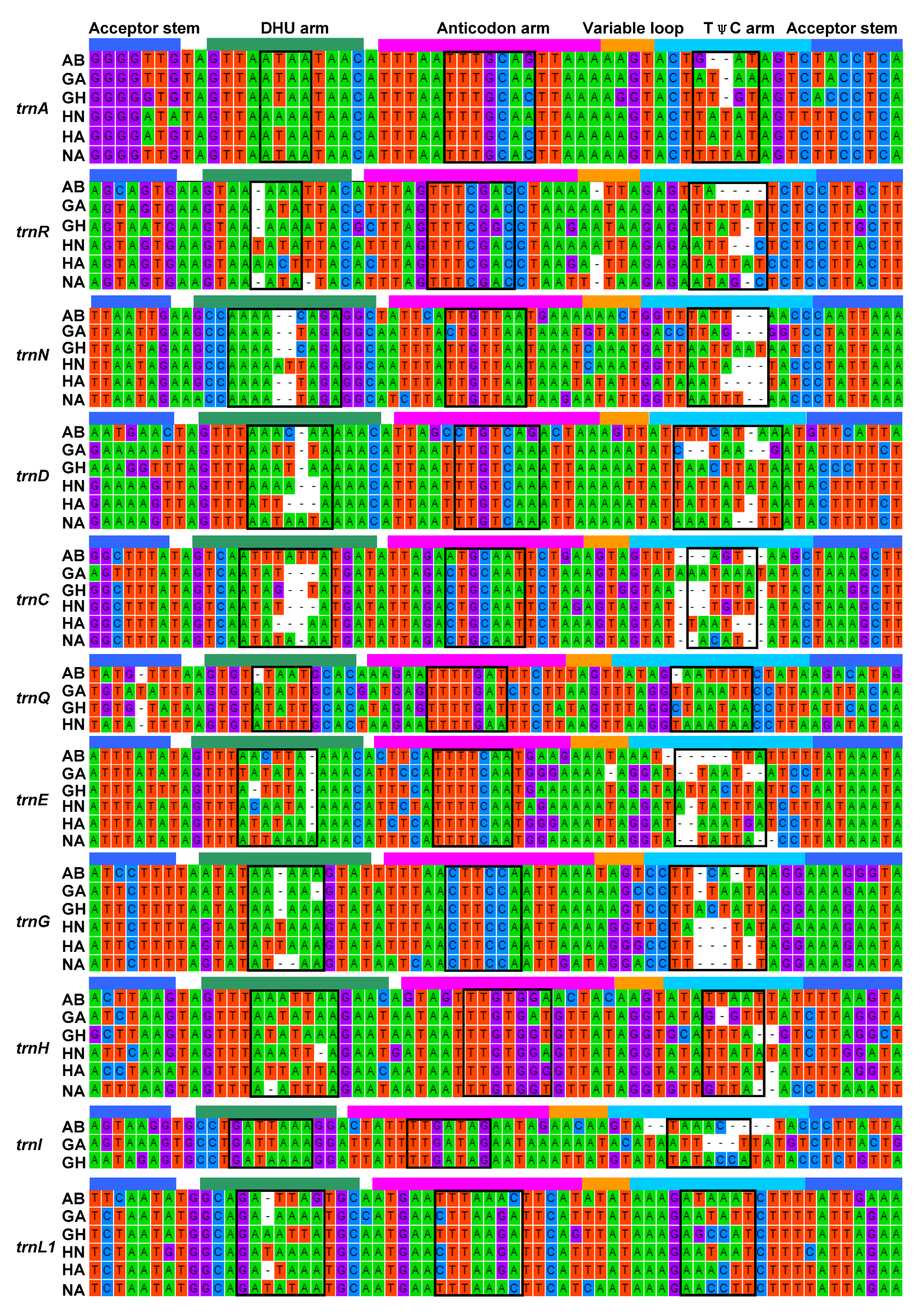

Supplement: Figure S4 — Alignment of tRNA families ( trnA - trnL1 ) in six nabid mtDNAs. The loop regions are highlighted by the black pane. (TIFF) [file pone.0045925.s004.tiff]

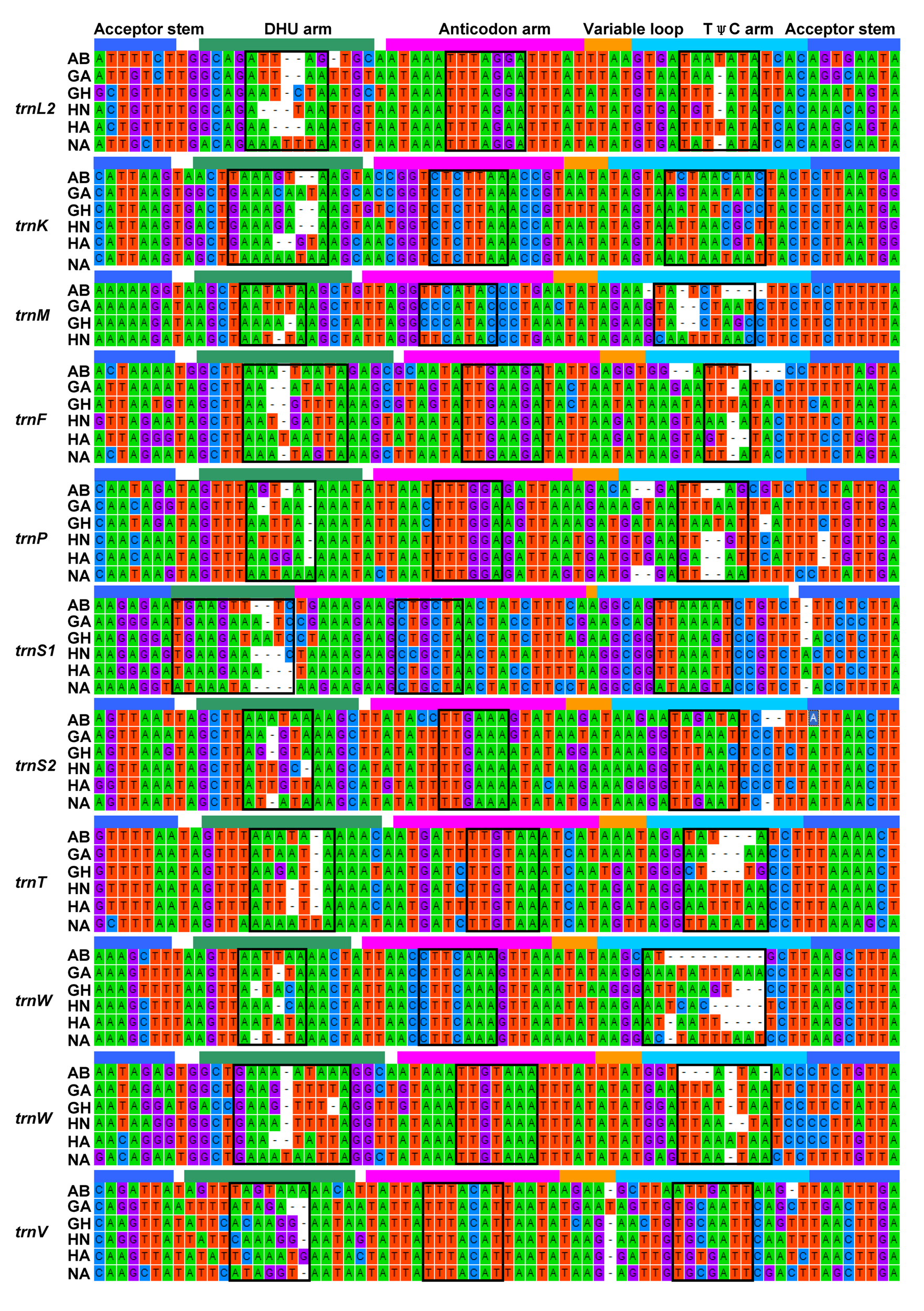

Supplement: Figure S5 — Alignment of tRNA families ( trnL2 - trnV ) in six nabid mtDNAs. The loop regions are highlighted by the black pane. (TIFF) [file pone.0045925.s005.tiff]

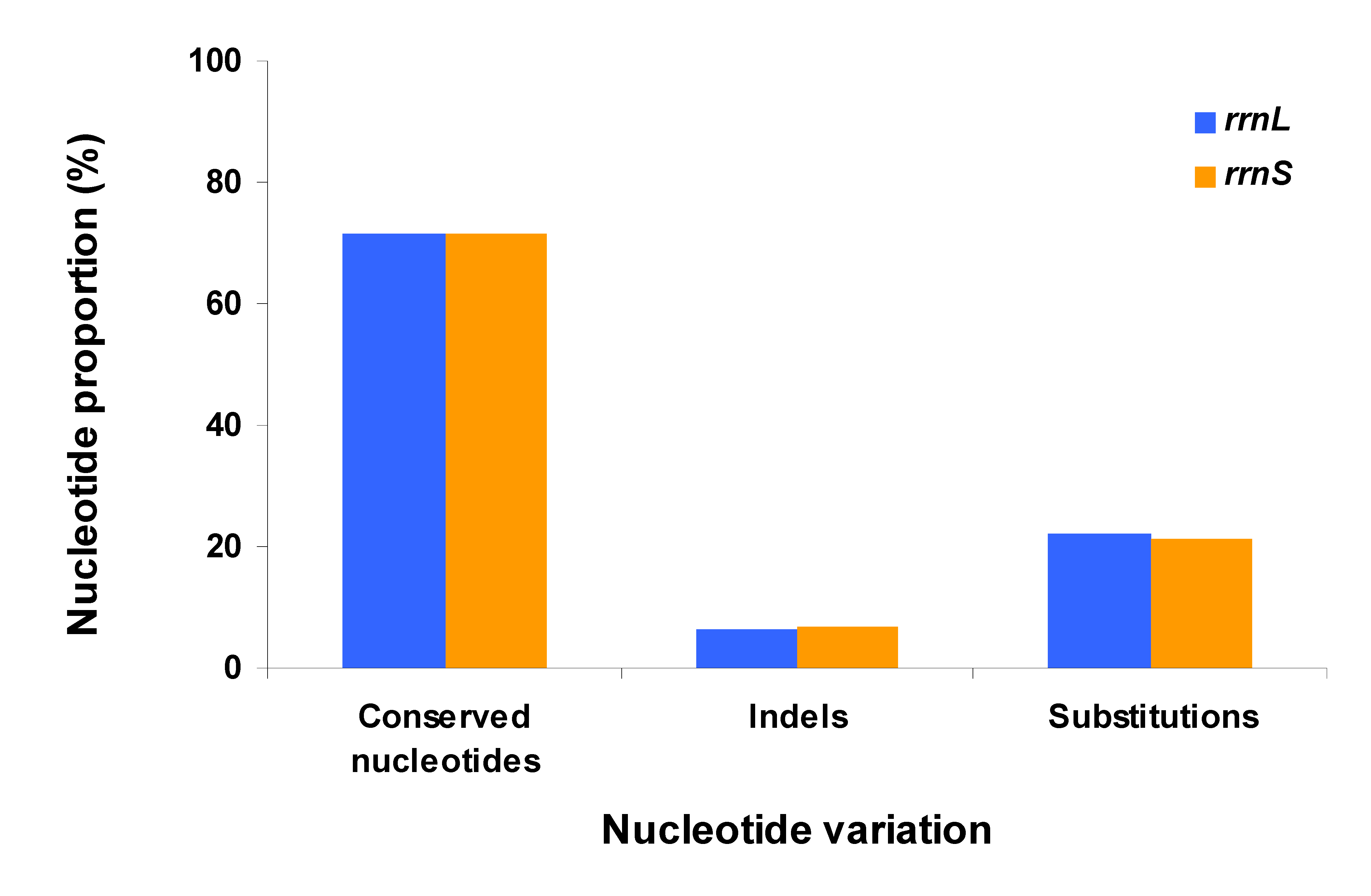

Supplement: Figure S6 — Nucleotide variation in six nabid mitochondrial rRNAs. (TIF) [file pone.0045925.s006.tif]

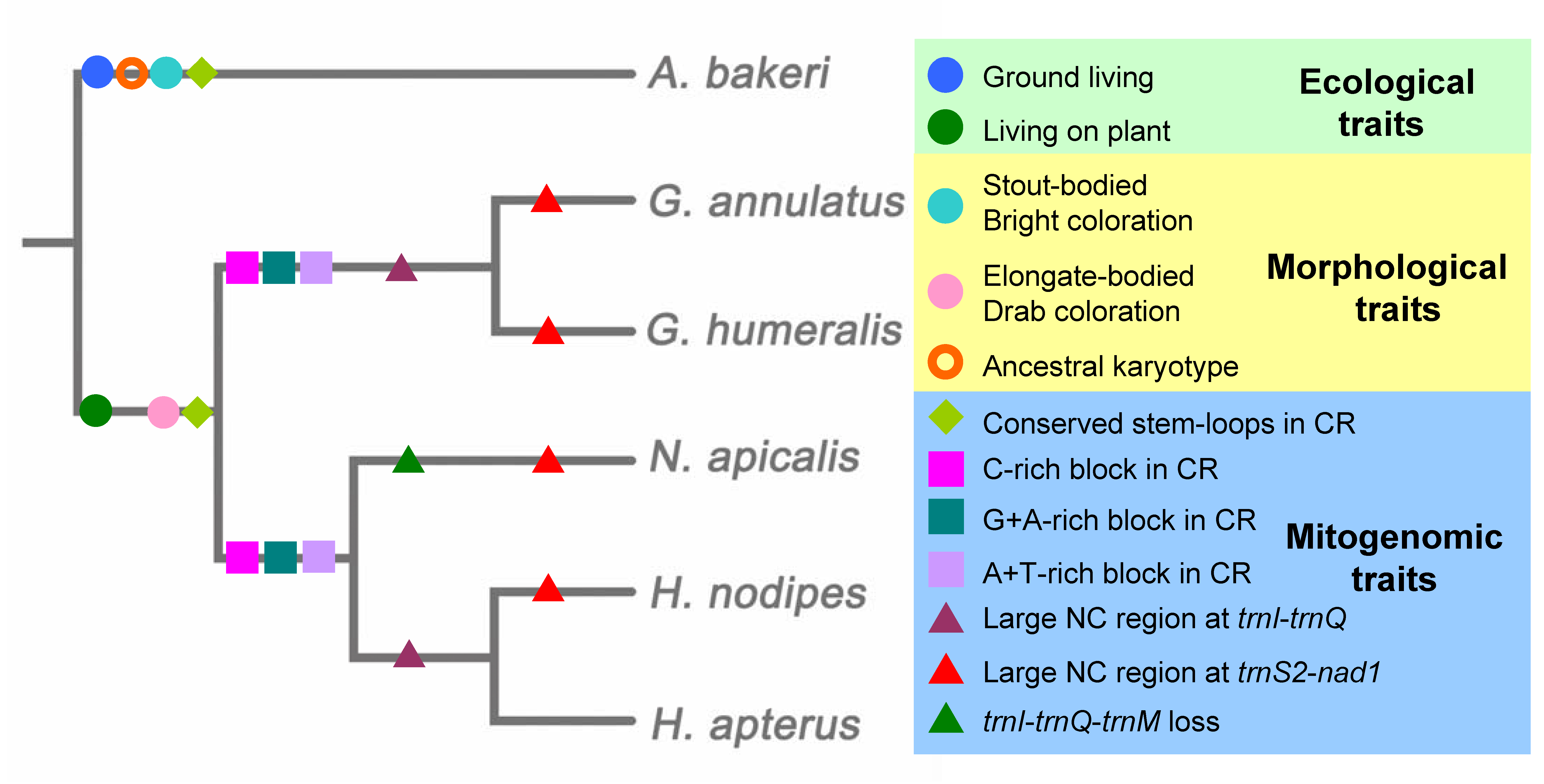

Supplement: Figure S7 — Mapping various traits onto the phylogenetic tree of six damsel bugs. Ecological, morphological and mitogenomic traits are mapped onto the phylogenetic tree inferred from the mitogenomic data. (TIF) [file pone.0045925.s007.tif]
