# Supplementary material for: Comparative Mitogenomic Analysis of Damsel Bugs Representing Three Tribes in the Family Nabidae (Insecta: Hemiptera)
Source: PLoS One. 2012 Sep 28;7(9):e45925. doi: 10.1371/journal.pone.0045925 (PMC3461043; doi:10.1371/journal.pone.0045925)
Supplement: Table S1 — Structural features of six nabid mitogenomes. (DOC) [file pone.0045925.s008.doc]

**Table S1 Structural features of** six nabid mitogenomes

| **mtDNA(bp)** | ***A. bakeri*** | ***N. apicalis*** | ***G. annulatus*** | ***G. humeralis*** | ***H. apterus**** | ***H. nodipes**** |
| --- | --- | --- | --- | --- | --- | --- |
| **Size** | 15, 851 | 15, 588 | 16, 660 | 18, 165 | 15, 995 | 16, 457 |
| **A+T%** | 73.5 | 74.9 | 75.5 | 72.6 | 76.9 | 76.4 |
| **CR** | 1, 312 | 1, 070 | 1, 189 | 1, 367 | >1, 554 | >1, 247 |
| ***rrnL*** | 1, 252 | 1, 289 | 1, 287 | 1, 276 | 1, 286 | 1, 287 |
| ***rrnS*** | 790 | 820 | 804 | 808 | 811 | 816 |
| ***cox1*** | 1, 534 | 1, 534 | 1, 539 | 1, 539 | 1, 539 | 1, 539 |
|  | (TTG/T-) | (TTG/T-) | (ATG/TAA) | (TTG/TAA) | (TTG/TAA) | (TTG/TAA) |
| ***cox2*** | 679 | 679 | 679 | 679 | 679 | 679 |
|  | (ATT/T-) | (ATA/T-) | (ATG/T-) | (ATG/T-) | (ATT/T-) | (ATC/T-) |
| ***cox3*** | 788 | 788 | 788 | 788 | 788 | 788 |
|  | (ATG/TA-) | (ATG/TA-) | (ATG/TA-) | (ATG/TA-) | (ATG/TA-) | (ATG/TA-) |
| ***cytb*** | 1, 137 | 1, 137 | 1, 137 | 1, 137 | 1, 137 | 1, 135 |
|  | (ATG/TAG) | (ATG/TAG) | (ATG/TAG) | (ATG/TAG) | (ATG/TAG) | (ATG/T-) |
| ***nad1*** | 922 | 921 | 918 | 922 | 924 | 924 |
|  | (ATT/T-) | (ATG/TAA) | (ATG/TAA) | (GTG/T-) | (ATG/TAA) | (ATG/TAA) |
| ***nad2*** | 999 | 981 | 999 | 993 | 1, 002 | 1, 005 |
|  | (ATT/TAA) | (ATC/TAA) | (ATT/TAA) | (ATT/TAA) | (ATT/TAA) | (ATT/TAA) |
| ***nad3*** | 354 | 354 | 354 | 354 | 354 | 354 |
|  | (ATA/TAA) | (ATT/TAA) | (ATA/TAA) | (ATT/TAA) | (ATA/TAA) | (ATT/TAA) |
| ***nad4*** | 1, 329 | 1, 322 | 1, 326 | 1, 330 | 1, 332 | 1, 332 |
|  | (ATG/TAA) | (ATG/TA-) | (ATG/TAA) | (ATG/T-) | (ATG/TAA) | (ATG/TAA) |
| ***nad4L*** | 294 | 294 | 303 | 291 | 294 | 291 |
|  | (ATT/TAG) | (ATC/TAA) | (ATT/TAA) | (ATA/TAA) | (ATT/TAA) | (ATA/TAA) |
| ***nad5*** | 1, 706 | 1, 700 | 1, 707 | 1, 701 | 1, 707 | 1, 707 |
|  | (ATT/TA-) | (ATA/TA-) | (ATT/TAA) | (ATT/TAG) | (ATA/TAA) | (ATT/TAA) |
| ***nad6*** | 498 | 504 | 504 | 504 | 504 | 504 |
|  | (ATA/TAA) | (ATA/TAA) | (ATA/TAA) | (ATA/TAA) | (ATA/TAA) | (ATA/TAA) |
| ***atp6*** | 684 | 684 | 669 | 684 | 684 | 684 |
|  | (ATG/TAA) | (ATG/TAG) | (ATG/TAA) | (ATG/TAG) | (ATG/TAA) | (ATG/TAA) |
| ***atp8*** | 159 | 159 | 159 | 159 | 159 | 159 |
|  | (ATA/TAA) | (ATA/TAA) | (ATC/TAA) | (ATA/TAA) | (ATA/TAA) | (ATA/TAA) |

"*”: nearly complete mitogenomes. Start and stop codons for PCGs are indicated in parentheses.
