# Supplementary material for: Comparative Mitogenomic Analysis of Damsel Bugs Representing Three Tribes in the Family Nabidae (Insecta: Hemiptera)
Source: PLoS One. 2012 Sep 28;7(9):e45925. doi: 10.1371/journal.pone.0045925 (PMC3461043; doi:10.1371/journal.pone.0045925)
Supplement: Table S2 — Codon distribution in six nabid mtDNAs. (DOC) [file pone.0045925.s009.doc]

**Table S2 Codon distribution in six nabid mtDNAs.**

| **Amino acid** | **Codon** | ***AB*** | ***NA*** | ***GA*** | ***GH*** | ***HA*** | ***HN*** | **Amino acid** | **Codon** | ***AB*** | ***NA*** | ***GA*** | ***GH*** | ***HA*** | ***HN*** |
| --- | --- | --- | --- | --- | --- | --- | --- | --- | --- | --- | --- | --- | --- | --- | --- |
| **Phe (F)** | **UUU** | **244** | **227** | **242** | **245** | **220** | **221** | **Tyr (Y)** | **UAU** | **137** | **170** | **167** | **149** | **180** | **169** |
|  | UUC | 63 | 46 | 52 | 35 | 62 | 51 |  | UAC | 28 | 24 | 31 | 34 | 12 | 33 |
| **Leu (L)** | **UUA** | **336** | **345** | **365** | **328** | **375** | **377** | **Stop (*)** | UAA |  |  |  |  |  |  |
|  | UUG | 49 | 46 | 33 | 52 | 34 | 32 |  | UAG |  |  |  |  |  |  |
|  | CUU | 52 | 50 | 55 | 60 | 34 | 39 | **His (H)** | **CAU** | **49** | **58** | **60** | **58** | **59** | **61** |
|  | CUC | 8 | 4 | 6 | 6 | 4 | 0 |  | CAC | 20 | 19 | 15 | 19 | 13 | 16 |
|  | CUA | 94 | 305 | 41 | 71 | 52 | 61 | **Gln (Q)** | **CAA** | **49** | **50** | **51** | **42** | **52** | **54** |
|  | CUG | 5 | 4 | 9 | 11 | 2 | 1 |  | CAG | 11 | 7 | 3 | 12 | 6 | 4 |
| **Ile (I)** | **AUU** | **310** | **305** | **336** | **297** | **361** | **341** | **Asn (N)** | **AAU** | **155** | **155** | **172** | **141** | **177** | **170** |
|  | AUC | 60 | 41 | 37 | 51 | 44 | 34 |  | AAC | 31 | 37 | 35 | 39 | 34 | 31 |
| **Met (M)** | **AUA** | **283** | **353** | **347** | **313** | **384** | **386** | **Lys (K)** | **AAA** | **70** | **78** | **81** | **76** | **81** | **79** |
|  | AUG | 36 | 37 | 37 | 50 | 22 | 32 |  | AAG | 23 | 18 | 16 | 20 | 12 | 19 |
| **Val(V)** | GUU | 78 | 65 | 74 | 67 | 50 | 48 | **Asp (D)** | **GAU** | **47** | **61** | **55** | **50** | **66** | **61** |
|  | GUC | 6 | 2 | 8 | 11 | 2 | 5 |  | GAC | 17 | 8 | 10 | 22 | 3 | 8 |
|  | **GUA** | **83** | **115** | **102** | **108** | **124** | **120** | **Glu (E)** | **GAA** | **69** | **64** | **62** | **62** | **69** | **61** |
|  | GUG | 17 | 11 | 15 | 25 | 2 | 10 |  | GAG | 19 | 12 | 11 | 14 | 11 | 15 |
| **Ser (S)** | **UCU** | **106** | **89** | **88** | **88** | 95 | 88 | **Cys (C)** | **UGU** | **39** | **46** | **43** | **42** | **42** | **43** |
|  | UCC | 30 | 21 | 17 | 34 | 9 | 8 |  | UGC | 8 | 8 | 4 | 13 | 6 | 7 |
|  | UCA | 97 | 86 | 83 | 81 | **96** | **91** | **Trp (W)** | **UGA** | **86** | **82** | **76** | **80** | **86** | **89** |
|  | UCG | 2 | 6 | 5 | 2 | 0 | 2 |  | UGG | 22 | 15 | 17 | 17 | 12 | 9 |
| **Pro (P)** | **CCU** | **60** | **63** | **73** | **59** | **63** | 56 | **Arg (R)** | CGU | 14 | 22 | 24 | 12 | 21 | **27** |
|  | CCC | 32 | 26 | 24 | 36 | 12 | 11 |  | CGC | 2 | 0 | 1 | 4 | 1 | 3 |
|  | CCA | 43 | 39 | 40 | 29 | 49 | **62** |  | **CGA** | **34** | **24** | **25** | **27** | **28** | 22 |
|  | CCG | 4 | 4 | 1 | 4 | 6 | 2 |  | CGG | 6 | 6 | 4 | 10 | 3 | 2 |
| **Thr (T)** | **ACU** | 70 | **96** | **80** | **79** | **91** | **90** | **Ser (S)** | AGU | 44 | 50 | 33 | 28 | 48 | 52 |
|  | ACC | 36 | 26 | 20 | 31 | 15 | 15 |  | AGC | 4 | 13 | 7 | 16 | 6 | 4 |
|  | ACA | **72** | 74 | 69 | 74 | 69 | 84 |  | **AGA** | **82** | **61** | **78** | **95** | **66** | **57** |
|  | ACG | 4 | 2 | 5 | 5 | 2 | 4 |  | AGG | 6 | 5 | 3 | 4 | 3 | 2 |
| **Ala (A)** | **GCU** | 47 | **60** | **58** | 46 | 44 | **58** | **Gly (G)** | GGU | 81 | 68 | 66 | 46 | 66 | **91** |
|  | GCC | 32 | 18 | 21 | 37 | 11 | 11 |  | GGC | 9 | 6 | 5 | 16 | 3 | 0 |
|  | GCA | **48** | 54 | 50 | **47** | **65** | 53 |  | **GGA** | **92** | **90** | **86** | **101** | **98** | 77 |
|  | GCG | 1 | 2 | 2 | 5 | 0 | 0 |  | GGG | 33 | 33 | 46 | 47 | 26 | 30 |

Values in bold type stand for the most commonly used codon for amino acid. Underlined stand for the cognate codon of tRNA for each amino acid.
