# Supplementary material for: Comparative Mitogenomic Analysis of Damsel Bugs Representing Three Tribes in the Family Nabidae (Insecta: Hemiptera)
Source: PLoS One. 2012 Sep 28;7(9):e45925. doi: 10.1371/journal.pone.0045925 (PMC3461043; doi:10.1371/journal.pone.0045925)
Supplement: Table S3 — Evolutionary rates of six nabid mitochondrial PCGs. (DOC) [file pone.0045925.s010.doc]

**Table S3 Evolutionary rates of six nabid mitochondrial PCGs.**

| **Gene** | **ka** | **ks** | **ka/ks** | **GC%** |
| --- | --- | --- | --- | --- |
| ***cox1*** | 0.06 | 0.89 | 0.07 | 0.32 |
| ***cox2*** | 0.12 | 0.96 | 0.12 | 0.28 |
| ***cox3*** | 0.12 | 0.90 | 0.14 | 0.29 |
| ***cytb*** | 0.12 | 1.05 | 0.11 | 0.28 |
| ***nad1*** | 0.16 | 0.88 | 0.18 | 0.26 |
| ***nad2*** | 0.36 | 0.71 | 0.50 | 0.20 |
| ***nad3*** | 0.21 | 0.99 | 0.22 | 0.24 |
| ***nad4*** | 0.27 | 0.70 | 0.38 | 0.23 |
| ***nad4L*** | 0.32 | 0.85 | 0.37 | 0.23 |
| ***nad5*** | 0.26 | 0.63 | 0.42 | 0.23 |
| ***nad6*** | 0.34 | 0.28 | 0.52 | 0.20 |
| ***atp6*** | 0.21 | 0.85 | 0.24 | 0.24 |
| ***atp8*** | 0.51 | 0.55 | 0.93 | 0.17 |
