# Supplementary material for: Comparative Mitogenomic Analysis of Damsel Bugs Representing Three Tribes in the Family Nabidae (Insecta: Hemiptera)
Source: PLoS One. 2012 Sep 28;7(9):e45925. doi: 10.1371/journal.pone.0045925 (PMC3461043; doi:10.1371/journal.pone.0045925)
Supplement: Table S4 — Statistics on NC sequences in six nabid mitogenomes. (DOC) [file pone.0045925.s011.doc]

**Table S4 Statistics on NC sequences in six** nabid mitogenomes

| **Species** | **NC (%)** | **Length (bp)** | **Large NC region (bp)** | | |
| --- | --- | --- | --- | --- | --- |
|  |  |  | ***trnI*-*trnQ*** | ***trnS2*-*nad1*** | ***rrnS*-*trnI (CR)*** |
| ***A. bakeri*** | 8.56 | 1, 357 | 3 | 20 | 1, 312 |
| ***G. annulatus*** | 12.38 | 2, 062 | 221 | 584 | 1, 189 |
| ***G. humeralis*** | 19.64 | 3, 567 | 1, 539 | 480 | 1, 367 |
| ***H. apterus**** | 9.94 | 1, 590 | - | 15 | >1, 554 |
| ***H. nodipes**** | 11.99 | 1, 974 | >539 | 93 | >1, 247 |
| ***N. apicalis*** | 7.7 | 1, 198 | - | 102 | 1, 070 |

"*”: nearly complete mitogenomes.
