# Supplementary material for: Comparative Mitogenomic Analysis of Damsel Bugs Representing Three Tribes in the Family Nabidae (Insecta: Hemiptera)
Source: PLoS One. 2012 Sep 28;7(9):e45925. doi: 10.1371/journal.pone.0045925 (PMC3461043; doi:10.1371/journal.pone.0045925)
Supplement: Table S5 — Occurrence of tandem repetitions in six nabid mtDNA NC regions. (DOC) [file pone.0045925.s012.doc]

**Table S5 Occurrence of tandem repetitions in six** nabid mtDNA NC region

| **Species** | **Size (bp) and Copy Number (cn)** | | |
| --- | --- | --- | --- |
|  | ***trnI*-*trnQ*** | ***trnS2*-*nad1*** | ***rrnS*-*trnI* (CR)** |
| ***A. bakeri*** | N/A | N/A | 580/6 |
| ***G. annulatus*** | N/A | 582/4 | N/A |
| ***G. humeralis*** | 1442/6 | 467/3 | N/A |
| ***H. apterus**** | N/A | N/A | 322/23 |
| ***H. nodipes**** | 540/4 | N/A | 84/4 |
| ***N. apicalis*** | N/A | N/A | 170/2 |

"*”: nearly complete mitogenomes; N/A, not applicable.
