# Supplementary material for: Comparative Mitogenomic Analysis of Damsel Bugs Representing Three Tribes in the Family Nabidae (Insecta: Hemiptera)
Source: PLoS One. 2012 Sep 28;7(9):e45925. doi: 10.1371/journal.pone.0045925 (PMC3461043; doi:10.1371/journal.pone.0045925)
Supplement: Table S6 — Information of five sequenced nabid species included in the present study. (DOC) [file pone.0045925.s013.doc]

**Table S6 Information of five sequenced nabid species included in the present study**

| **Species** | **Locality** | **Time** | **Voucher** |
| --- | --- | --- | --- |
| *Gorpis annulatus* | Jianfengling, Hainan | 2007-5-11 | Vhem-00106 |
| *Gorpis humeralis* | Zhaotong, Yunnan | 2009-9-12 | Vhem-00102 |
| *Himacerus apterus* | Shennongjia, Hubei | 2009-7-12 | Vhem-00103 |
| *Himacerus nodipes* | Zhaotong, Yunnan | 2009-9-16 | Vhem-00105 |
| *Nabis apicalis* | Wuyishan, Fujian | 2009-707 | Vhem-00104 |
