# Supplementary material for: Comparative Mitogenomic Analysis of Damsel Bugs Representing Three Tribes in the Family Nabidae (Insecta: Hemiptera)
Source: PLoS One. 2012 Sep 28;7(9):e45925. doi: 10.1371/journal.pone.0045925 (PMC3461043; doi:10.1371/journal.pone.0045925)
Supplement: Table S7 — Primer sequences used in this study. (DOC) [file pone.0045925.s014.doc]

**Table S7** Primer sequences used in this study

| **No. fragment** | **Primer ID** | **Nucleotide sequence (5’-3’)** | **Reference** |
| --- | --- | --- | --- |
| **1** | TW- J1301 | GTTAAWTAAACTAATARCCTTCAAA | Simon *et al.*, 2006 |
|  | C1- N1738 | TTTATTCGTGGRAATGCYATRTC | Simon *et al.*, 2006 |
| **2** | C1-J1709 | AATTGGWGGWTTYGGAAAYTG | Simon *et al.*, 2006 |
|  | C1- N2776 | GGTAATCAGAGTATCGWCGNGG | Simon *et al.*, 2006 |
| **3** | C1-J2756 | ACATTTTTTCCTCAACATTT | Simon *et al.*, 2006 |
|  | C2-N3665 | CCACAAATTTCTGAACACTG | Simon *et al.*, 2006 |
| **4** | F-3665 | GATGCAATTCCTGGACGACTAAACCA | Present study |
|  | R-7795 | TCCATTTTCTTCTTGACTACCAGCAGC | Present study |
| **5** | N5-J7572 | AAAGGGAATTTGAGCTCTTTTWGT | Simon *et al.*, 2006 |
|  | N4-N8727 | AAATCTTTRATTGCTTATTCWTC | Simon *et al.*, 2006 |
| **6** | N4-J8641 | CCAGAAGAACACAAACCATG | Simon *et al.*, 2006 |
|  | N4L-N9629 | GTTTGTGAGGGTGCAATAGG | Simon *et al.*, 2006 |
| **7** | F-9629 | CAACATGAGCCTTGGGTAAC | Present study |
|  | R-9648 | ATTTTGCGAATTGGGTTATT | Present study |
| **8** | N4L-J9648 | TCCCAACACACCTTCACAAAC | Simon *et al.*, 2006 |
|  | CB- N11010 | TATCAACAGCAAATCCTCCTCA | Simon *et al.*, 2006 |
| **9** | CB-J10621 | CTCATACTGATGAAATTTTGGTTC | Simon *et al.*, 2006 |
|  | CB-N11526 | TTCTACTGGTCGTGCTCCAATTCA | Simon *et al.*, 2006 |
| **10** | F-11526 | TGCAATTTTACGATCCATTC | Present study |
|  | R-12888 | GTTACCTAAGGGATAACAGCGT | Present study |
| **11** | LR-J12888 | CCGGTCTGAACTCAGATCATGTA | Simon *et al.*, 2006 |
|  | LR-N13889 | ATTTATTGTACCTTTTGTATCAG | Simon *et al.*, 2006 |
| **12** | LR-J13342 | CCTTAGCACAGTTAAAATACTGC | Simon *et al.*, 2006 |
|  | LR-N14220 | TTATGCACATATCGCCCGTC | Simon *et al.*, 2006 |
| **13** | LR-J14197 | GTAAAYCTACTTTGTTACGACTT | Simon *et al.*, 2006 |
|  | SR-N14745 | GTGCCAGCAAYCGCGGTTATAC | Simon *et al.*, 2006 |
| **14** | SR- J14610 | ATAATAGGGTATCTAATCCTAGT | Simon *et al.*, 2006 |
|  | TM- N200 | ACCTTTATAARTGGGGTATGARCC | Simon *et al.*, 2006 |
| **15** | F-200 | AGATGCCTGATTAAAGGATTA | Present study |
|  | R-1301 | AAGATGGCTGAGTAAGGTTAT | Present study |
